# Cardiopulmonary Exercise Testing in Patients With Interstitial Lung Disease

**DOI:** 10.3389/fphys.2020.00832

**Published:** 2020-07-10

**Authors:** Yannick Molgat-Seon, Michele R. Schaeffer, Christopher J. Ryerson, Jordan A. Guenette

**Affiliations:** ^1^Department of Kinesiology and Applied Health, University of Winnipeg, Winnipeg, MB, Canada; ^2^Centre for Heart Lung Innovation, St. Paul’s Hospital, The University of British Columbia, Vancouver, BC, Canada; ^3^Department of Physical Therapy, Faculty of Medicine, The University of British Columbia, Vancouver, BC, Canada; ^4^Division of Respiratory Medicine, Faculty of Medicine, The University of British Columbia, Vancouver, BC, Canada

**Keywords:** dyspnea, exercise capacity, hypoxemia, idiopathic pulmonary fibrosis, ventilatory limitation

## Abstract

Interstitial lung disease (ILD) is a heterogeneous group of conditions characterized by fibrosis and/or inflammation of the lung parenchyma. The pathogenesis of ILD consistently results in exertional dyspnea and exercise intolerance. Cardiopulmonary exercise testing (CPET) provides important information concerning the pathophysiology of ILD that can help inform patient management. Despite the purported benefits of CPET, its clinical utility in ILD is not well defined; however, there is a growing body of evidence that provides insight into the potential value of CPET in ILD. Characteristic responses to CPET in patients with ILD include exercise-induced arterial hypoxemia, an exaggerated ventilatory response, a rapid and shallow breathing pattern, critically low inspiratory reserve volume, and elevated sensations of dyspnea and leg discomfort. CPET is used in ILD to determine cause(s) of symptoms such as exertional dyspnea, evaluate functional capacity, inform exercise prescription, and determine the effects of pharmacological and non-pharmacological interventions on exercise capacity and exertional symptoms. However, preliminary evidence suggests that CPET in ILD may also provide valuable prognostic information and can be used to ascertain the degree of exercise-induced pulmonary hypertension. Despite these recent advances, additional research is required to confirm the utility of CPET in patients with ILD. This brief review outlines the clinical utility of CPET in patients with ILD. Typical patterns of response are described and practical issues concerning CPET interpretation in ILD are addressed. Additionally, important unanswered questions relating to the clinical utility of CPET in the assessment, prognostication, and management of patients with ILD are identified.

## Introduction

The term interstitial lung disease (ILD) refers to a large, heterogenous group of conditions that involve variable degrees of fibrosis and/or inflammation of the lung parenchyma (King, 2005). Although the etiology varies across ILD subtypes, the pathogenesis of ILD leads to a host of physiological abnormalities including progressive reductions in lung volumes, pulmonary gas exchange limitations (Chetta et al., 2004; Young and Bye, 2011), decreased cardiovascular function (Panagiotou et al., 2017) as well as skeletal muscle atrophy and dysfunction (Panagiotou et al., 2016). The pathophysiological features of ILD consistently result in an increased perception of dyspnea, particularly during physical exertion, and reduced exercise tolerance (Collard and Pantilat, 2008; Holland, 2010). Cardiopulmonary exercise testing (CPET) is often considered to be the gold-standard for evaluating exertional dyspnea and exercise intolerance in patients with cardiorespiratory conditions, and provides important information that complements diagnostic investigations performed at rest (Palange et al., 2007). Despite the well-documented application of CPET in other forms of cardiorespiratory disease (American Thoracic and American College of Chest, 2003), its utility in ILD is not well characterized (Bonini and Fiorenzano, 2017); however, a growing body of evidence relating to the pathophysiological responses to exercise in patients with ILD provides new insight into the potential value of CPET in this population.

In this review, we provide a brief summary of recent findings concerning the clinical utility of CPET in patients with ILD. Typical patterns of response and how they differ from those observed in health are described and practical issues concerning the interpretation of CPET variables in ILD are addressed. Given that the focus of this review is on recent findings related to CPET in ILD, the interested reader is directed elsewhere for detailed descriptions of foundational concepts including the pathophysiology of ILD (Parker et al., 2011; Bagnato and Harari, 2015) and the physiological responses to exercise in patients with ILD (Lama and Martinez, 2004; Molgat-Seon et al., 2019).

### Physiological Responses to CPET

#### Functional Capacity

The primary pathophysiological features of ILD have a detrimental impact on the integrative physiological response to exercise. Patients with ILD have reduced functional capacity when compared to healthy individuals, as evidenced by a lower peak power output and oxygen uptake during incremental cycle exercise testing (Wehr and Johnson, 1976; Burdon et al., 1983) as well as a shorter distance achieved during 6-min walk testing (Chang et al., 1999). As is the case in other forms of cardiorespiratory disease, performing CPET in patients with ILD provides a direct assessment of their functional capacity, however, the mechanisms of exercise intolerance in ILD are complex, multifactorial, and variable across patients and ILD sub-types (Holland, 2010; Young and Bye, 2011). The restrictive ventilatory impairment and decreased pulmonary gas exchange efficiency negatively impact the ventilatory response to exercise, while pulmonary vascular destruction and arterial hypoxemia impair the cardiovascular response to exercise. Additionally, ILD may involve skeletal muscle atrophy and dysfunction, which would further decrease exercise capacity (Panagiotou et al., 2016). The abovementioned factors may act individually or synergistically to reduce exercise tolerance (Holland, 2010; Young and Bye, 2011). It is therefore challenging to determine the exact cause of exercise limitation in a given ILD patient. Nevertheless, monitoring the ventilatory, cardiovascular, skeletal muscle, and sensory responses to exercise during CPET in ILD reveals common patterns that differ from those observed in health and can enable clinicians to determine the primary cause(s) of symptom limitation (Table 1).

**TABLE 1:** CPET responses in healthy individuals and how they differ in patients with ILD.

CPET measure	Healthy individuals	ILD
**Functional capacity**		
V̇O_2_ max, ml⋅kg^–1^⋅min^–1^	Typically >85% predicted	Reduced, often <85% predicted
Peak Power Output, W	Typically >85% predicted	Reduced, often <85% predicted
Anaerobic Threshold, %V̇O_2_ max	Typically >40% of predicted V̇O_2_ max	Reduced, often <40% predicted V̇O_2_ max
**Ventilatory and pulmonary gas exchange**		
*f*_B_, breaths⋅min^–1^	Typically <50 breaths⋅min^–1^ throughout exercise	Increased for a given power output or V̇_E_, can be >50 breaths⋅min^–1^ at peak exercise
V_T_, l	Typically increases to up 50–60% VC and plateaus	Typically increases to 50–60% VC and plateaus but is reduced for any given V̇_E_
V_D_/V_T_	Decreases relative to rest, typically <0.30 at peak exercise	Minimal change from rest and can increase during exercise to >0.30
V̇_E_/V̇CO_2_	Typically <32–34 at or near anaerobic threshold and <36 at peak	Elevated throughout exercise, typically >32–34 at anaerobic threshold and >36 at peak
V̇_E_/MVV, %	Typically <85% throughout exercise	Variable, but can be >85% at peak exercise
SaO_2_	Preserved throughout exercise, similar to resting values	Variable, but typically decrease below resting values
**Cardiovascular**		
HR, % predicted maximum	Typically >90% of age predicted values at peak exercise	Variable, but will often be below age predicted values at peak exercise
V̇O_2_/HR, ml⋅beat^–1^	Typically >80% of predicted at peak exercise	Reduced, usually <80% of predicted at peak exercise
**Symptoms**		
Dyspnea	Increases progressively during exercise, usually >5 at peak exercise	Increased for a given power output and V̇_E_, usually >5 at peak exercise
Leg discomfort	Increases progressively during exercise, usually >5 at peak exercise	Increased for a given power output, usually >5 at peak exercise
**Invasive measures**		
EMG_di_, % of maximum	Increases progressively during exercise up to ∼60–70% of maximum	Increased for a given power output and V̇_E_, similar at peak exercise
PaO_2_, mmHg	Relatively stable during exercise, typically <10 mmHg reduction from rest	Typically drops progressively during exercise, >10 mmHg reduction from rest
P(A-a)O_2_, mmHg	Increases progressively during exercise, <25 mmHg at peak exercise	Increases progressively during exercise, often >30 mmHg at peak exercise
mPAP– Q̇ slope, mmHg⋅min^–1^⋅l^–1^	<3 mmHg⋅min^–1^⋅l^–1^	Variable, but can be >3 mmHg⋅min^–1^⋅l^–1^

*EMG_di_, diaphragm electromyography; f_B_, breathing frequency; HR, heart rate; mPAP, mean pulmonary artery pressure; MVV, maximal voluntary ventilation; PaO_2_, partial pressure of arterial oxygen; P(A-a)O_2_, alveolar-arterial partial pressure of oxygen gradient; Q̇, cardiac output; SaO_2_, arterial oxygen saturation; V̇_E_, minute ventilation; V̇_E_/V̇CO_2_, ventilatory equivalent for carbon dioxide; V_D_, dead space volume; V̇O_2_, oxygen uptake; V_T_, tidal volume.*

#### Ventilatory and Pulmonary Gas Exchange Responses

During incremental exercise, minute ventilation (V̇_E_) must increase in order to meet the demands associated with the rising oxygen uptake (V̇O_2_) and carbon dioxide production (V̇CO_2_). Similarly, the increase in V̇O_2_ necessitates a greater rate of oxygen diffusion into the blood in order to maintain arterial oxygen saturation (SaO_2_), thereby ensuring adequate oxygen delivery to exercising muscles. In health, the exercise-induced increase in V̇_E_ is primarily achieved via an expansion of tidal volume (V_T_) until it reaches ∼60% of vital capacity (VC). Further increases in V̇_E_ are then achieved by increasing breathing frequency (*f*_B_). The aforementioned breathing pattern is considered optimal as it minimizes the work of breathing and dead-space ventilation. In patients with ILD, vital capacity (VC) is reduced, which limits their ability to expand V_T_ and by association, reduces their maximum voluntary ventilation (MVV) (Faisal et al., 2016). Moreover, their lung compliance is decreased, which correspondingly increases the mechanical and metabolic cost of breathing for a given V̇_E_ (Faisal et al., 2016). For patients with ILD, the higher mechanical and metabolic cost of breathing during exercise may impair ventricular performance, due to the large intrathoracic pressure swings, and may increase the fraction of total cardiac output directed to the respiratory muscles at the expense of locomotor muscle blood flow (Dominelli et al., 2017); however, direct evidence supporting this notion in patients with ILD is currently lacking. Together, the reductions in VC, MVV, and lung compliance alter the ventilatory response to exercise in ILD. At rest, patients with ILD typically have a rapid breathing pattern (i.e., normal V_T_ and high *f*_B_). During incremental exercise, patients with ILD will initially increase V̇_E_ by expanding V_T_ in a similar manner as observed in healthy individuals but will reach a plateau in V_T_ at a much lower V̇_E_ and exercise intensity due to their reduced VC. Subsequent increases in V̇_E_ are then achieved by further increasing *f*_B_. This rapid and shallow breathing pattern implies that the ratio of dead-space volume to V_T_ (V_D_/V_T_) is typically high during exercise in patients with ILD. At rest and during exercise at a given absolute intensity, V̇_E_ is also increased in ILD due to ventilation-perfusion mismatch (Jernudd-Wilhelmsson et al., 1986) as well as increased ventilatory drive resulting from arterial hypoxemia and an early-onset of metabolic acidosis (Van Meerhaeghe et al., 1981), which is evidenced by an elevated V̇_E_/V̇CO_2_ throughout exercise. Thus, some patients with ILD will experience ventilatory limitation during exercise (Marciniuk et al., 1994b), which can be crudely assessed based on whether the ratio of V̇_E_ and MVV is greater than 85%; however, it is noteworthy that in many cases, patients with ILD will terminate exercise despite having adequate ventilatory reserve (Marciniuk et al., 1994a; Faisal et al., 2016). Lastly, the pathological decrement in pulmonary gas exchange at rest is worsened during exercise, thereby resulting in a progressive decrease in SaO_2_, even in those who are not hypoxemic at rest (Young and Bye, 2011). Exercise-induced arterial hypoxemia is a characteristic feature of patients with ILD (Agusti et al., 1988). In fact, patients with ILD often exhibit a greater degree of arterial desaturation during exercise than patients with chronic obstructive pulmonary disease (Du Plessis et al., 2017). The importance of assessing the degree of exercise-induced arterial hypoxemia during CPET is highlighted by its association with disease severity and prognosis (Lama et al., 2003; Vainshelboim et al., 2016). Given the limitations associated with pulse oximetry, CPET involving serial assessments of arterial blood gases may be warranted if the main purpose of the test is to determine the adequacy of pulmonary gas exchange (American Thoracic and American College of Chest, 2003). Arterial blood gas sampling enables the assessment of several important parameters, including the partial pressure of arterial oxygen (PaO_2_) and carbon dioxide (PaCO_2_), the alveolar-arterial partial pressure of oxygen gradient [P(A-a)O_2_], as well as direct measures of SaO_2_ and V_D_/V_T_. The assessment of P(A-a)O_2_, which is characteristically widened during exercise in ILD (Agusti et al., 1991) is particularly informative given its correlation with the degree of fibrosis and cellularity obtained from a lung biopsy, as well as its potential prognostic value, at least in idiopathic pulmonary fibrosis (IPF) (Fulmer et al., 1979; Agusti et al., 1994).

#### Cardiovascular Responses

Despite primarily affecting the lungs, many forms of ILD involve obliteration of the pulmonary capillary bed and remodeling of the pulmonary vasculature (Nathan et al., 2007; Seeger et al., 2013). Depending on the degree of pulmonary vascular involvement, some patients with ILD will have impairments in cardiovascular function that contribute to exercise limitation. In healthy individuals performing incremental exercise, there is a linear increase in cardiac output (Q̇) as a function of V̇O_2_. The exercise-induced increase in Q̇ is achieved via a progressive increase in heart rate (HR) and an increase in stroke volume up to approximately 50–60% of maximal oxygen uptake (V̇O_2_ max), at which point SV plateaus. Patients with ILD are generally capable of increasing Q̇ in proportion to V̇O_2_ during exercise at relatively low intensities; however, at higher exercise intensities, some patients have a diminished rate of increase in Q̇ (Bush and Busst, 1988; Degani-Costa et al., 2015), which is presumably due to a reduction in stroke volume (Degani-Costa et al., 2015). Indeed, patients with ILD typically have a lower oxygen pulse, an indirect marker of stroke volume, during exercise than healthy individuals (Faisal et al., 2016). Thus, patients with ILD often have an abnormal HR response to exercise, whereby HR is higher for a given absolute exercise intensity when compared to their healthy counterparts (Baughman et al., 1984), which may be due to constraints on stroke volume, deconditioning, or both. At peak exercise, HR values in patients with ILD are variable since some patients fail to reach their age-predicted maximum HR due to cessation of exercise prior to reaching their physiological maxima (Faisal et al., 2016), whereas others may have a critically low HR reserve (Schwaiblmair et al., 1996; Baughman et al., 2011). During standard CPET, measures of HR, electrocardiogram parameters, and non-invasive measures of arterial blood pressure are the only available direct cardiovascular measures. However, in cases where there is a high index of suspicion for exercise-induced pulmonary hypertension despite negative tests at rest, invasive CPET with right heart catherization may be warranted in order to directly evaluate the pulmonary vascular response to exercise (Maron et al., 2013). In such cases, some patients may exhibit an exaggerated pulmonary artery pressure response to exercise, whereby a given increase in Q̇ results in a greater increase in mean pulmonary artery pressure than in healthy individuals (Degani-Costa et al., 2015). Alternatively, echocardiography may be employed as a non-invasive alternative to cardiac catherization for the assessment of exercise-induced pulmonary hypertension (Himelman et al., 1989; Reichenberger et al., 2009; D’Alto et al., 2011), although echocardiographic data obtained during exercise in patients with ILD is relatively limited.

#### Skeletal Muscle Responses

It has been suggested that skeletal muscle atrophy and dysfunction may be an important systemic consequence of ILD (Panagiotou et al., 2016). Indeed, the strength of the quadriceps muscles is reduced in patients with ILD when compared to predicted values, and is significantly associated with functional capacity (Nishiyama et al., 2005; Watanabe et al., 2013). If present, skeletal muscle dysfunction would accelerate the onset of metabolic acidosis and skeletal muscle fatigue during exercise. Although standard CPET parameters are not directly indicative of skeletal muscle dysfunction, early termination of a test due to intolerable leg discomfort in the face of significant ventilatory and/or cardiovascular reserves may indicate locomotor muscle dysfunction (Marciniuk et al., 1994a; Nishiyama et al., 2005).

#### Sensory Responses

For patients with ILD, dyspnea is the most common symptom, particularly upon exertion (Collard and Pantilat, 2008). CPET is often employed to evaluate the mechanisms of unexplained dyspnea in patients with cardiorespiratory disease, including those with ILD (Bonini and Fiorenzano, 2017). During incremental exercise, patients with ILD report higher levels of dyspnea than healthy individuals for a given absolute exercise intensity or V̇_E_ (O’Donnell et al., 1998; Faisal et al., 2016). The increased dyspnea is thought to reflect the awareness of an increased neural respiratory drive necessitated by the aforementioned pathophysiological alterations in respiratory mechanics and pulmonary gas exchange efficiency (Schaeffer et al., 2018). This notion is supported by the fact that when patients with ILD are given supplemental oxygen during constant-load exercise, the neural respiratory drive to the diaphragm decreases along with their perception of dyspnea (Schaeffer et al., 2017b). Neural respiratory drive to the diaphragm can be estimated during CPET using esophageal electromyography (Faisal et al., 2016; Schaeffer et al., 2018).

Given that ILD primarily affects the lungs, it is logical to assume that ventilatory factors would lead to the increased perception of exertional dyspnea. Indeed, those who experience ventilatory limitation during CPET are more likely to discontinue exercise because of intolerable dyspnea; however, this may not always be the case (Marciniuk et al., 1994a). Patients with ILD who do not exhibit signs of overt ventilatory limitation typically terminate exercise due to intolerable leg discomfort (Marciniuk et al., 1994a), which may be due to attendant hypoxemia, skeletal muscle dysfunction, and/or cardiovascular limitation (Hansen and Wasserman, 1996; Panagiotou et al., 2016). Regardless of the root cause, patients with ILD experience exertional dyspnea and elevated perceptions of leg discomfort during CPET at relatively low exercise intensities. It follows that carefully assessing the perceptual responses to CPET, at rest and throughout exercise, provides important information that can be contextualized with ventilatory, cardiovascular, and metabolic measurements to gain insight regarding the cause of the aforementioned sensations. Additional assessments such as asking patients to report the reason they stopped exercising (i.e., due to dyspnea, leg discomfort, or due to another reason) and evaluating the qualitative descriptors of dyspnea using a validated questionnaire may be helpful in ascertaining the underlying cause of symptom limitation during CPET in ILD (O’Donnell et al., 1998).

### Clinical Utility of CPET

It is well established that CPET is a valuable tool for the assessment of patients with several forms of cardiorespiratory disease (Arena and Sietsema, 2011; Bonini and Fiorenzano, 2017). Yet, CPET currently lacks a defined role in ILD management (Bradley et al., 2008; Raghu et al., 2011; Assayag et al., 2018). Nevertheless, recent findings highlight the potential benefits that could be derived from CPET in patients with ILD. Herein, we summarize our current understanding of the clinical utility of CPET in patients with ILD, highlight important findings that outline how the use of CPET in ILD could be expanded, and identify areas where additional research is required.

#### Current Use of CPET

ILD diagnosis is established by a multidisciplinary team of respirologists, chest radiologists, and lung pathologists based on a combination of clinical information derived from pulmonary function tests, chest imaging, and lung tissue histology (Richeldi et al., 2019). CPET data are not a requirement for a multidisciplinary diagnosis of ILD but may be used to inform patient management. Indeed, there are several reasons for conducting CPET in patients with ILD (Table 2; American Thoracic and American College of Chest, 2003). Its most obvious and well-established application is in assessing the cause(s) of symptoms such as exertional dyspnea, and objectively evaluating functional capacity (Mezzani, 2017). Determining the intensity at which desaturation occurs may be useful for the prescription of ambulatory oxygen therapy (Palange et al., 2007). Additionally, pulmonary rehabilitation is advocated for some patients with ILD (Assayag et al., 2018) and CPET can be used as a baseline assessment of functional capacity prior to enrollment as well as for precise exercise prescription (Bernard et al., 2014). Repeated CPET also allows clinicians to assess the change in exercise tolerance and exertional symptoms following pulmonary rehabilitation (Tonelli et al., 2017) or other pharmacological and non-pharmacological interventions (Jackson et al., 2010; Blanco et al., 2011; Schaeffer et al., 2017b). Unlike other exercise tests (e.g., 6-min walk test), CPET has the added advantage of being able to identify the physiological mechanisms of improvement. However, it is noteworthy that the minimal clinically-important difference (MCID) for CPET parameters has not yet been established in ILD, which complicates the longitudinal assessment of CPET data.

**TABLE 2:** Primary reasons for conducting CPET in patients with ILD.

Reasons for conducting CPET
• Determining the cause(s) of exertional dyspnea
• Assessing functional capacity and the mechanism(s) of exercise intolerance
• Determining the magnitude of exercise-induced hypoxemia
• Establishing a baseline prior to and setting exercise training intensity for pulmonary rehabilitation
• Evaluating the acute and chronic response to pharmacological and non-pharmacological interventions on exercise performance, symptoms, and physiology
• Evaluating pulmonary gas exchange abnormalities
• Patient prognostication
• Determining the presence of co-morbidities
• Evaluation for lung transplantation

#### Important Considerations When Conducting CPET

As is the case with other forms of cardiorespiratory disease, the clinical utility of CPET in ILD depends on the variables measured, the exercise modality used, and how results are interpreted. CPET provides a substantial amount of data that can be used to inform patient management. Depending on the indications for CPET in patients with ILD (Table 2), several additional measures may be added to the standard, obligatory measures (i.e., V̇O_2_, V̇CO_2_, V̇_E_, HR, etc.) in order to achieve a particular goal. For example, if the intent is to evaluate the degree of respiratory limitation to exercise, having the patient perform inspiratory capacity maneuvers during exercise may be warranted (Guenette et al., 2013) along with flow-volume loop analyses. Similarly, comprehensively investigating decrements in pulmonary gas exchange efficiency may require arterial blood gas sampling, while determining the degree of pulmonary vascular impairment may necessitate right heart-catherization or echocardiography. In situations where repeated CPET is required, constant-load exercise, rather than incremental exercise, is most sensitive for detecting changes in key physiological and perceptual variables (Puente-Maestu et al., 2016). It is also important to compare sensory and physiological data between repeat CPETs at standardized submaximal measurement times rather than focusing exclusively on peak exercise responses (Schaeffer et al., 2017a). Regardless of the indication for CPET, it is important to define the aim of the test and tailor the method of testing accordingly.

#### Potential Application of CPET

In comparison to other forms of cardiorespiratory disease, the application of CPET in ILD has been relatively limited (American Thoracic and American College of Chest, 2003; Bonini and Fiorenzano, 2017). However, recent evidence highlights promising areas where pulmonary gas exchange parameters obtained during CPET could improve the management of patients with ILD (Bonini and Fiorenzano, 2017). Pulmonary gas exchange measures during CPET have been used to non-invasively determine whether patients with ILD have pulmonary hypertension. Specifically, patients with ILD who had resting pulmonary hypertension that was confirmed by right heart catheterization had significantly lower partial pressure of end-tidal carbon dioxide and mixed expired carbon dioxide during incremental exercise than those who had no evidence of resting pulmonary hypertension (Armstrong et al., 2013). Thus, CPET may be helpful in revealing possible co-morbidities such as pulmonary hypertension. Another area that has garnered interest is the utility of CPET data to inform ILD prognosis. Pulmonary gas exchange parameters obtained during CPET appear to be superior to resting pulmonary function tests for the prognostication of ILD (Keogh and Crystal, 1980; Miller et al., 1995) and PaO_2_ at peak exercise is a significant predictor of survival in IPF (King et al., 2001). The utility of CPET for ILD prognostication may have important implications for the referral of patients for lung transplants (Layton et al., 2017) given that the allocation of lungs is primarily based on 1-year mortality risk (McCurry et al., 2009). However, the notion that CPET parameters have value in the prognostication of ILD remains controversial and requires additional research (American Thoracic and American College of Chest, 2003).

#### Challenges Associated With CPET Interpretation

The interpretation of CPET data in patients with ILD is challenging for several reasons. First, similar pathological responses may occur in several other forms of disease (e.g., chronic obstructive pulmonary disease, pulmonary hypertension, and chronic heart failure) (Marciniuk and Gallagher, 1994) with these conditions also frequently occurring in patients with ILD. Second, CPET responses can differ substantially across patients given the variable etiology, symptoms, and prognosis of the various ILD subtypes. Finally, in certain cases, patients with ILD may terminate exercise due to factors that are unrelated to their underlying lung disease, such as physical deconditioning or malingering (Marciniuk and Gallagher, 1994). Therefore, it is critical that CPET responses be interpreted in the context of all available information, including whether or not physiological maxima was reached during testing (Younes, 1984).

#### Considerations for Future Research

The widespread use of 6 min walk tests in ILD highlights the value of using exercise to gain information that can be used to inform the management of patients with ILD (du Bois et al., 2011). Given the host of additional data obtained, it stands to reason that CPET may be even more valuable from a clinical perspective despite its resource intensive nature. Indeed, the potential uses of CPET in ILD are numerous, but additional research is required to promote its widespread use. Most notably, establishing the MCID for CPET parameters in ILD is critical. Future studies should also consider the variability in exercise responses between ILD subtypes and how these differences might provide information to improve patient management. Particular focus should be placed on pulmonary gas exchange responses as they appear to be the most sensitive to the pathophysiological changes associated with ILD.

## Summary and Conclusion

The pathophysiology of ILD has a negative impact on the ventilatory, cardiovascular, and skeletal muscle responses to exercise, thereby leading to exertional dyspnea and reduced exercise capacity. CPET is an excellent tool for assessing the severity of exertional dyspnea and mechanisms of exercise limitation in patients with various forms of cardiorespiratory disease; however, its application in ILD has remained limited. A growing body of evidence supports the use of CPET in patients with ILD. CPET is currently used to assess functional capacity, to inform exercise prescription, and to evaluate the effects of various interventions. Recent findings highlight that, in addition to its current uses, CPET-derived measures may help improve the management of patients with ILD. Despite these recent advances, additional research is required in order to confirm the utility of CPET in this population.

## Author Contributions

All authors contributed to drafting and critically revising the manuscript, approved the final version of the manuscript, and take responsibility for the integrity of its content.

## Conflict of Interest

The authors declare that the research was conducted in the absence of any commercial or financial relationships that could be construed as a potential conflict of interest.
